# Banana Blossom (*Musa acuminate* Colla) Incorporated Experimental Diets Modulate Serum Cholesterol and Serum Glucose Level in Wistar Rats Fed with Cholesterol

**DOI:** 10.1155/2016/9747412

**Published:** 2016-11-30

**Authors:** Ruvini Liyanage, Saranya Gunasegaram, Rizliya Visvanathan, Chathuni Jayathilake, Pabodha Weththasinghe, Barana Chaminda Jayawardana, Janak Kamil Vidanarachchi

**Affiliations:** ^1^National Institute of Fundamental Studies, Hantana Road, Kandy, Sri Lanka; ^2^Department of Animal Science, Faculty of Agriculture, University of Peradeniya, Peradeniya, Sri Lanka

## Abstract

Hypocholesterolaemic and hypoglycaemic effect of banana blossom were studied in high-cholesterol fed rats. Experimental groups were fed for 4 weeks, with casein as the basal diet (CN), in comparison with two diets containing 0.5% cholesterol (CD) and 0.5% cholesterol + 21% banana blossom powder (CDB). Serum total cholesterol, non-HDL-cholesterol level, and serum glucose concentrations were lower in CDB fed group compared with CD fed group. Lower serum cholesterol and glucose level (*P* < 0.05) in CDB fed group were followed by higher faecal weight, caecal weight, caecal* Lactobacilli*, and* Bifidobacteria* population in CDB fed group compared to CD diet fed group. Lower serum AST level in banana blossom fed rats showed the reduction in oxidative stress induced by high cholesterol diet. Based on these data, it could be speculated that banana blossom incorporated experimental diets may modulate the hypocholesterolaemic and hypoglycaemic responses in Wistar rats.

## 1. Introduction

Blossom of the banana plant (*Musa acuminata* Colla), by-product of banana cultivation, is often consumed as a vegetable in many Asian countries such as Sri Lanka, Malaysia, Indonesia, and the Philippines [1]. In Sri Lanka, it is consumed as a curry as well as a boiled or deep fried salad with rice and wheat bread [2]. Banana blossoms have tremendous nutritional value and are rich source of dietary fibre and some biologically active compounds like vitamin C, tannin, myoinositol phosphates, and alpha tocopherol [3–5]. High levels of dietary fibre intake are associated with significantly lower prevalence rates for coronary heart disease, stroke, and peripheral vascular disease [6–8]. However, average fibre intakes for children and adults are alarmingly less than that of the recommended level [9]. Food components with antioxidant properties may prevent cardiovascular diseases by inhibiting the oxidative damage to LDL-cholesterol [10]. Recently, polyphenols have been found to affect blood lipids in animals in a similar manner as soluble dietary fibre [11]. In another study, chloroform, water, and ethanol extract of another banana variety (*Musa sapientum*) flowers were found to exhibit hypoglycaemic activity in alloxan diabetic rat [12–14]. Hemicellulose fraction of* Musa sapientum* showed high amounts of total polyphenols and total antioxidants [15], indicating that banana blossom is a rich source of dietary fibre associated with polyphenols, which could promote health benefits. As the dietary fibre consumption is alarmingly low, it is important to investigate the potential of underutilized vegetables as potent antioxidants and blood lipid lowering agents. Though banana blossom has been in the Sri Lankan diet for years, no research has been done to investigate the health potential of banana blossoms as a fibre and antioxidant rich vegetable. Thus, the aim of this study was to investigate the banana blossom incorporated experimental diets on blood lipids and blood glucose level in rats fed cholesterol.

## 2. Materials and Methods

### 2.1. Animals and Diets

Fifteen male Wistar rats (7 weeks old) were randomly assigned to three groups of 5 each (Medical Research Institute, Sri Lanka). All rats were individually housed in plastic cages. The animal facility was maintained on a 12 h light-dark cycle at a temperature of 23 ± 1°C and relative humidity of 60 ± 5%. The composition of each diet is shown in Table 1. The experimental groups were fed for 4 weeks, with casein as the basal diet (CN), in comparison with two diets containing 0.5% cholesterol (CD) and 0.5% cholesterol + 21% banana blossom powder (CDB). The rats were allowed free access to food and water for 4-week experimental period. Body weight and food consumption were recorded weekly and daily, respectively. The blood samples (1 mL) were collected at the beginning and at the end of the experimental period from jugular veins of fasting rats anaesthetized by sodium pentobarbital. The samples were taken into tubes without any anticoagulant. After the samples were allowed to stand at room temperature for 2 h, the serum was separated by centrifugation at 1500 ×g for 20 min. At the end of the 4-week experimental period, all faeces excreted during last 3 days were collected. The rats were anaesthetized with sodium pentobarbital and killed, and the livers and caecum were quickly removed, washed with cold saline (9 g NaCl/L), blotted dry on filter paper, and weighed before freezing for storage.

This experimental design was approved by the Animal Experiment Committee of Faculty of Veterinary Medicine and Animal Science, University of Peradeniya. All animal procedures conformed to standard principles described in* Guide for the Care and Use of Laboratory Animals* [16].

### 2.2. Proximate Composition, Polyphenol Content, and Antioxidant Activity of Banana Blossom

Proximate composition of dried banana blossoms was done according to AOAC method [17]. The antioxidant activity of banana blossoms was determined by the DPPH free radical scavenging assay [18]. Polyphenol content in banana blossoms was measured by Folin- Ciocalteu method [19].

### 2.3. Serum Lipids, Glucose, AST Level, and Antioxidant Activity

Total cholesterol (TC), HDL cholesterol (HDL-C), triglyceride (TG), Aspartate amino transferase (AST), and glucose concentrations in the serum were determined enzymatically using commercially available reagent kits (ProDia Internationals, Germany). The non-HDL-cholesterol concentration was calculated as follows: [non-HDL-C] = [TC] − [HDL-C]. Serum antioxidant actvity was measured by Ferric Reducing Antioxidant Power (FRAP) assay [20].

### 2.4. Growth of Bacteria in the Caecum

Coliform, anaerobes,* Lactobacillus*, and* Bifidobacterium* from the caecum were inoculated and incubated for 3 days on MacConkey agar (Oxoid), GAM agar (Nissui), Rogosa agar (Nissui), and Bifidobacterium agar (Himedia, India) at 37°C by the GasPak method described previously [21].

### 2.5. Statistical Analysis

Data are presented as the mean and standard deviation for serum TC, HDL-C, non-HDL-C, TG, and glucose level at the prescribed times. Completely randomized design was conducted and data were analyzed by one-way analysis of variance (ANOVA) using the General Linear Model (GLM) procedure of SAS (SAS Institute Inc., 2000) software program. The significance of differences among means was separated by Duncan's multiple range test (SAS Institute, Cary, NC, USA). Differences were considered significant at *P* < 0.05.

## 3. Results

### 3.1. Proximate Composition and Antioxidant Activity of Banana Blossom

Crude fat, crude fibre, crude protein, ash, carbohydrate, and dry matter content of banana blossoms were 6.54, 23.71, 12.58, 18.30, 34.36, and 65.49% respectively. Antioxidant activity of banana blossom was 21.02 ± 0.31 and the value was expressed as IC_50_ values (ppm) against the corresponding standard ascorbic acid. Polyphenol content in banana blossoms was 12.02 ± 0.5 mg/g (DW).

### 3.2. Body Weight, Food Intake, and Liver, Caecal, and Faecal Weight

There was no difference in the initial body weight, food intake, and liver weight among the groups (Table 2). Body weight in the CDB incorporated diet fed group was lower (*P* < 0.05) than CD fed group and no difference was observed compared to CN fed group at the end of the experimental period. Caecal weight was high (*P* < 0.05) in CDB fed group compared to CD fed group. Faecal weight was high (*P* < 0.05) in CDB fed group compared to other two groups (Table 3).

### 3.3. Serum Lipid and Glucose Levels and Serum AST Level

**Figure 1:**
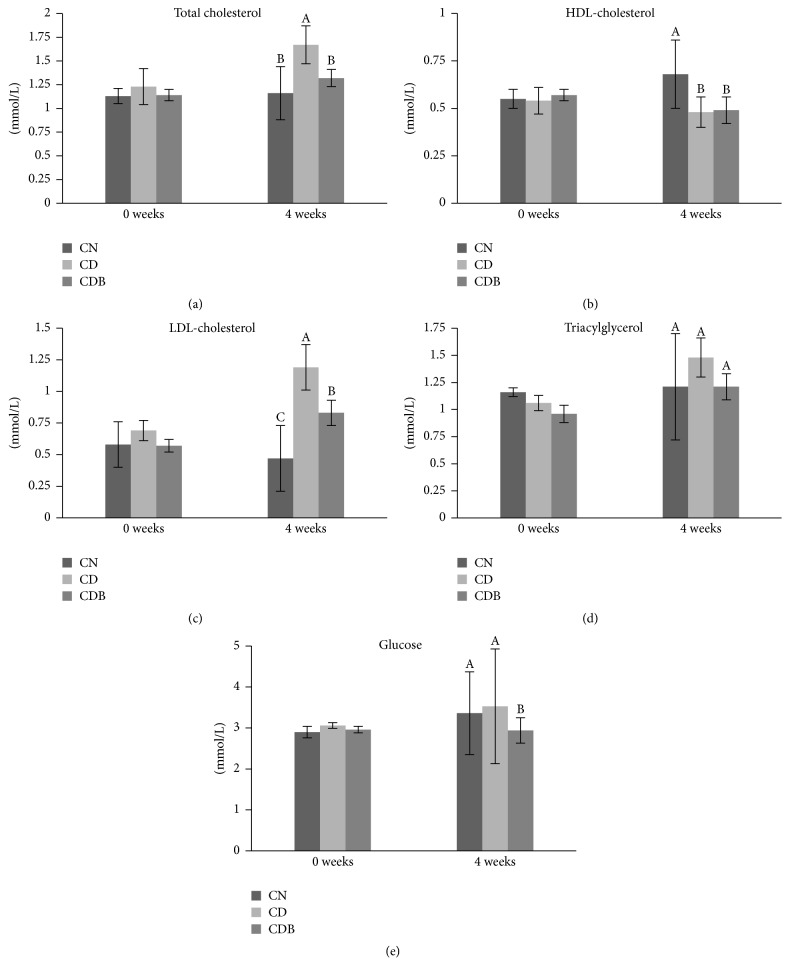
(a)–(e) Serum total cholesterol, high density-lipoprotein-cholesterol (HDL cholesterol), Non-HDL-cholesterol and triacylglycerol concentrations in rats fed on experimental diets for 4 weeks. Means followed by the same letters in a graph are not significant (*P* > 0.05). No significant differences were observed in 0 weeks.

Serum TC concentration was lower (*P* < 0.05) in CDB fed group compared to CD fed group and banana blossom (21%) in CDB diet countered the increase in total cholestreol induced by 0.5% cholesterol (Figure 1). Serum HDL-C level was lower (*P* < 0.05) in both CD and CDB fed groups compared to that in CN fed group. Serum Non-HDL-C level was lower (*P* < 0.05) in both CN and CDB fed groups compared to CD fed group. There was no difference in serum triacylglycerol level among 3 experimental diets fed groups. Serum glucose level was lower (*P* < 0.05) in CDB fed group compared to the other two groups. Serum AST level was high (*P* < 0.05) in CD fed group compared to the other two groups and there was no significant difference in serum antioxidant activity of the groups (Table 4).

### 3.4. Caecal Bacterial Population

Caecal* Lactobacilli* and* Bifidobacterium* population were higher (*P* < 0.05) in CDB fed group than the other two groups (Table 5). Caecal Coliform population was lower (*P* < 0.05) in CDB fed group than that in CN and CD fed groups. There was no difference in caecal anaerobic bacterial population among groups.

## 4. Discussion

In the present study, the effects of fibre and polyphenol rich banana blossom on serum cholesterol and serum glucose in rats fed a cholesterol enriched diet were examined [22]. Banana blossom diet (CDB) reduced the body weight in rats compared to cholesterol enriched control diet (CD) fed group and may support the previous findings that fibrous diet help reducing body weight [23, 24]. Lower serum total cholesterol and non-HDL-C level in CDB fed group were supported by higher crude fibre content in banana blossoms and faecal weight in rats fed CDB diet and were in agreement with previous studies showing that dietary fibre in experimental diets modulates serum cholesteol level in rats [23] and a reduction in non HDL-C in banana blossom supplemented diet fed group may be useful as a therapeutic treatment. Banana blossom has reduced high cholesterol diet induced serum AST level showing that banana blossom has a hepatoprotective ability by reducing oxidative stress. These data were further supported by observed higher antioxidant actvity and polyphenol content in banana blossoms. From a previous study done on the same banana species it was shown that banana blossom is a rich source of dietary fibre, unsaturated fatty acids, vitamin E, total saponins, and flavonoids [5]. These data suggested that polyphenol rich dietary fibres in banana blossom may modulate the lipid metabolism in rats [15]. In this way, several studies have suggested that antioxidant rich dietary fibre may have a positive effect on cardiovascular disease risk factors [25, 26].

Lower serum total and non HDL-C level in rats fed CDB group were further supported by higher caecal weight,* Bifidobacterium*, and* Lacobacilli* population suggesting that dietary fibre present in banana blossoms may exert a serum cholesterol-lowering effect through caecal fermentation and agrees with previous studies [27, 28]. It has been suggested that a higher proportion of butyrate in caecal content stimulates the movement of digesta, hence promoting faecal bulking and transit time [29]. Propionate, a fermentable metabolite of soluble fibre, is one of the prime components known to decrease the serum and hepatic cholesterol. Propionate concentrations at 1–1.25 mmol/L resulted in inhibition of cholestreol synthesis* in vitro* [30]. Supplementation of banana blossoms in CDB diet has reduced serum glucose level by 16.7% and this was in agreement with a previous study showing that chloroform, water, and ethanol extract of* Musa sapientum* flowers were found to exhibit hypoglycaemic activity in alloxan diabetic rat [12–14]. Phenolic compounds from various sources have been reported to prevent LDL oxidation* in vitro* and show marked hypolipidaemic activity* in vivo*, suggesting the effectiveness of polyphenols for the prevention and treatment of atherosclerosis [31, 32].

Limitations to this study include lack of mRNA data related to lipid and glucose metabaolism and lack of data on liver cholesterol level, caecal fatty acid composition, and faecal sterol level. Despite the limitations, this study demonstrates that banana blossom has the potential to reduce serum cholesterol and glucose level in rats. However, further research is needed to investigate the effect of diet containing banana blossoms on humans to elucidate the mechanism of action.

## Figures and Tables

**Table 1:** Composition of experimental diets (AIN 93G purified rodents diet).

Ingredients (g/kg diet)	CN	CD	CDB
Casein	210	210	183.5
Soy oil	100	100	86
Mineral mix	35	35	35
Vitamin mix	10	10	10
Cellulose	50	50	0
Corn starch	493	486.75	366.25
Cholesterol	0	5	5
Choline chloride	2	2	2
Sodium cholate	0	1.25	1.25
Sucrose	100	100	100
Dried banana blossom powder	0	0	211

Control diet (CN), 0.5% cholesterol diet (CD), and 0.5% cholesterol diet with 21% banana blossom (CDB).

**Table 2:** Initial body weight, final body weight, and feed intake of rats fed on experimental diets for 4 weeks.

Treatments	Initial body weight (g)	Final body weight (g)	Initial feed intake (g)	Final feed intake (g)
CN	200.0 ± 23.69^a^	295.8 ± 21.55^b^	16.2 ± 2.86^a^	13.6 ± 3.13^a^
CD	201.6 ± 19.47^a^	339.6 ± 19.75^a^	18.0 ± 3.80^a^	16.6 ± 3.36^a^
CDB	202.8 ± 27.12^a^	302.2 ± 29.02^b^	18.4 ± 5.36^a^	17.4 ± 3.64^a^

Values are expressed as mean ± standard deviation (SD). Mean values within a column with different superscript letters are significantly different (*P* < 0.05).

**Table 3:** Caecal, liver, and faecal weight of rats fed experimental diets.

	CN	CD	CDB
Liver (g)	2.62 ± 0.26^b^	3.65 ± 0.20^a^	3.44 ± 0.27^a^
Caecum (g)	0.59 ± 0.12^a^	0.49 ± 0.03^b^	0.61 ± 0.05^a^
Fecal weight (g)	0.92 ± 0.17^b^	0.72 ± 0.05^b^	3.72 ± 0.25^a^

Values are expressed as mean ± SD. Mean values within a row with different superscript letters were significantly different (*P* < 0.05).

**Table 4:** Serum antioxidant activity and serum AST (Aspartate amino transferase) level in rats fed experimental diets for 4 weeks.

Treatment	CN	CD	CDB
Antioxidant activity (AOA *μ*mol/L)	600.31 ± 78.61^a^	437.11 ± 98.01^a^	573.90 ± 191.38^a^
AST (ΔA/min)	1.07 ± 0.31^b^	6.40 ± 3.98^a^	0.74 ± 0.42^b^

Values are expressed as mean ± SD. Mean values within a row with different superscript letters were significantly different (*P* < 0.05).

**Table 5:** Caecal bacterial population (log⁡10 cfu/g content) in rats fed experimental diets for 4 weeks.

	CN	CD	CDB
Coliform	5.98 ± 0.26^a^	6.68 ± 0.20^a^	5.04 ± 0.27^b^
Lactic acid bacteria	7.09 ± 0.12^b^	7.10 ± 0.03^b^	7.91 ± 0.05^a^
Bifidobacteria	7.02 ± 0.17^b^	6.82 ± 0.05^b^	8.32 ± 0.25^a^
Total anaerobes	8.87 ± 0.17^a^	8.82 ± 0.05^a^	9.02 ± 0.25^a^

Values are expressed as mean ± SD. Mean values within a row with different superscript letters were significantly different (*P* < 0.05).
